# Alpinia oxyphylla Miq. fruit extract activates IGFR-PI3K/Akt signaling to induce Schwann cell proliferation and sciatic nerve regeneration

**DOI:** 10.1186/s12906-017-1695-2

**Published:** 2017-03-31

**Authors:** Yung-Ming Chang, Hen-Hong Chang, Chin-Chuan Tsai, Hung-Jen Lin, Tsung-Jung Ho, Chi-Xin Ye, Ping-Ling Chiu, Yueh-Sheng Chen, Ray-Jade Chen, Chih-Yang Huang, Chien-Chung Lin

**Affiliations:** 1 The School of Chinese Medicine for Post-Baccalaureate, I-Shou University, Kaohsiung, 840 Taiwan; 2 Chinese Medicine Department E-DA Hospital, Kaohsiung, 824 Taiwan; 3 1PT Biotechnology Co., Ltd., Taichung, 433 Taiwan; 4 Research Center for Chinese Medicine & Acupuncture, China Medical University, Taichung, 404 Taiwan; 5 Departments of Chinese Medicine, China Medical University Hospital, Taichung, 404 Taiwan; 6 School of Post-Baccalaureate Chinese Medicine, College of Chinese Medicine, China Medical University, Taichung, 404 Taiwan; 7 Division of Chinese Internal Medicine, China Medical University Hospital, Taichung, 404 Taiwan; 8 School of Post-Baccalaureate Chinese Medicine, China Medical University, Taichung, 404 Taiwan; 9 School of Chinese Medicine, China Medical University, Taichung, Taiwan; 10 Chinese Medicine Department, China Medical University Beigang Hospital, Beigang, Yunlin 651 Taiwan; 11 Graduate Institute of Basic Medical Science, China Medical University, No 91, Hsueh-Shih Road, Taichung, 404 Taiwan; 12 Department of Surgery, School of Medicine, College of Medicine, Taipei Medical University, Taipei, Taiwan; 13 Department of Health and Nutrition Biotechnology, Asia University, Taichung, Taiwan; 14 Orthopaedic Department Armed Forces General Hospital, Taichung, Taiwan

**Keywords:** *Alpinia oxyphylla* Miq. fruit extract, IGF-1, nerve regeneration, proliferation, RSC96 Schwann cell

## Abstract

**Background:**

It is known that the medicinal herb Alpinia oxyphylla Miq. is widely used as a remedy for diarrhea as well as the symptoms accompanying hypertension and cerebrovascular disorders. Moreover, it has also been reported that Alpinia oxyphylla Miq. has beneficial effects on anti-senescence and neuro-protection. This study focuses on the molecular mechanisms by which the Alpinia oxyphylla Miq. fruits promote neuron regeneration.

**Methods:**

A piece of silicone rubber was guided across a 15 mm gap in the sciatic nerve of a rat. This nerve gap was then filled with various doses of Alpinia oxyphylla Miq. fruits to assess their regenerative effect on damaged nerves. Further, we investigated the role of Alpinia oxyphylla Miq. fruits in RSC96 Schwann cell proliferation.

**Results:**

Our current results showed that treatment with the extract of Alpinia oxyphylla Miq. fruits triggers the phosphorylated insulin-like growth factor-1 receptor- phosphatidylinositol 3-kinase/serine-threonine kinase pathway, and up-regulated the proliferating cell nuclear antigen in a dose-dependent manner. Cell cycle analysis on RSC96 Schwann cells showed that, after exposure to Alpinia oxyphylla Miq. fruit extract, the transition from the first gap phase to the synthesis phase occurs in 12–18 h. The expression of the cell cycle regulatory proteins cyclin D1, cyclin E and cyclin A increased in a dose-dependent manner. Transfection with a small interfering RNA blocked the expression of phosphatidylinositol 3-kinase and induced down-regulation both on the mRNA and protein levels, which resulted in a reduction of the expression of the survival factor B-cell lymphoma 2.

**Conclusion:**

We provide positive results that demonstrate that Alpinia oxyphylla Miq. fruits facilitate the survival and proliferation of RSC96 cells via insulin-like growth factor-1 signaling.

## Background

It is known that the different anatomical structures of neurons and their regenerative abilities contribute to the central and peripheral nervous systems. Neuron injury stimulates various physiological responses that facilitate nerve cell regeneration. The neurons of the central nervous system in mammals lack the myelin sheath and therefore are incapable of regeneration. However, the neurons of the peripheral nervous system, which are surrounded by a myelin sheath, have the potential of regeneration and repair [1]. The ability of neuron regeneration is a result of intrinsic neuronal activities and other associated components, such as the Schwann cells. Schwann cells are capable of dedifferentiation, migration, proliferation, the expression of growth-promoting factors and the myelination of regenerating axons. After injury, the Schwann cells from the periphery migrate to the injured site to facilitate the repair processes [2, 3]. The growth factors that are produced by Schwann cells play an important role in peripheral nerve repair. Therefore, enhancing Schwann cell proliferation might be a potential approach for neuron regeneration in neuron injury. On the other hand, insulin-like growth factor-1 (IGF-1) has been characterized as a biochemical marker that is secreted in response to growth hormone to stimulate tissue growth [4]. IGF-1 modulates muscle satellite cells to undergo activation, proliferation and differentiation, leading to muscle regeneration and hypertrophy [5].

Biomaterials in combination with Chinese herbal medicine have been effectively used in nerve regeneration-related research. A silicon rubber chamber filled with Schwann cells has been shown to repair and bridge a 15 mm length of abrasion in rat sciatic nerves [6]. Therefore, treating Schwann cells with Chinese herbal medicines to enhance their therapeutic potential in guiding neuron regrowth is considered a possible approach to treat nerve injury.

Alpinate Oxyphyllae Fructus (*Alpinia oxyphylla* Miq., AOF) is a medicinal plant that is often used for treating ulcerations, gastralgia, diarrhea, dementia and tumors. Moreover, it has also been reported that AOF extracts exhibit potential neuro-protective effects against oxidative damage or neurotoxicity [7–12]. In our previous study, we evaluated whether AOF promotes RSC96 Schwann cell migration by ERK1/2, JNK and p38 signaling [13]. However, the beneficial effect of AOF on the nerve growth and regeneration facilitated by Schwann cells remains unclear.

In the present study, we compared the effects of AOF on Schwann cell proliferation and neuron regeneration in both in vivo and in vitro experiments. We examined the neuro-regenerative effect of AOF in animal models by injecting 0, 30, 60, 100, 150 or 200 mg/mL/kg concentrations of AOF into rat sciatic nerves, and in the in vitro model, we treated Schwann cells with 0, 20, 40, 60, 80, 100, 150 or 200 μg/mL of AOF.

## Methods

### AOF preparation

Fragments of AOF for research were purchased from the Shin-Long Pharmaceutical Company (Taichung, Taiwan, ROC). The AOF extract was prepared by boiling 150 g of AOF fragments in 600 mL of distilled water. The extract was filtered and the filtrate was concentrated under reduced pressure, and then stored at 4 °C until required. Finally, the AOF powdered extract was produced by spray drying.

### Animal model and treatments

The surgery was performed as previously described [14]. Thirty-six healthy adult Sprague-Dawley rats (220 ± 20 g), were allocated randomly to six experimental groups (*n* = 6 each) and housed in silicone chambers. Each chamber housed no more than 3 rats. The first group was the control group, and the chambers had a lumen volume of 25.5 μL, which administered saline only. The AOF groups 2–6 were administered AOF at 30, 60, 100, 150, and 200 mg/mL/kg, respectively.

All animals were housed in a standard housing room with chow and water available ad libitum. The housing room was maintained at 22 °C and 45% humidity, and kept under a regular 12 h light/dark cycle with lights on from 08:00 am to 20:00 pm.

Four weeks later, the animals were safely anesthetized using isoflurane, and the chamber connecting the nerve ends was re-exposed to examine the 15 mm gap within the chamber for evidence of successful nerve regeneration. The Institutional Animal Care and Use Committee (IACUC) of the China Medical University approved the protocols of animal use for experiment, and all animals maintained in the facilities were treated according to the principles of laboratory animal care (NIH publication).

### Cell culture and treatments

RSC96 Schwann cells were obtained from the American Type Culture Collection (ATCC, GA, USA) and were cultured in Dulbecco’s modified Eagle’s medium (DMEM) modified to contain 100 U/mL penicillin, 100 μg/mL streptomycin, 10% fetal bovine serum (FBS), 25 mM glucose, 4 mM L-glutamate and 1500 mg/L sodium bicarbonate at 37 °C under a humidified atmosphere of 5% CO_2_ and 95% air. After a 4-h period of serum-free culture, the RSC96 cells were treated with the indicated concentrations of AOF for 16–24 h, and then harvested for further analysis.

### Flow cytometry

The cells were harvested and washed twice with phosphate buffered saline (PBS, pH 7.2), then fixed in 500 μL 70% (*v*/v) ice-cold ethanol at 4 °C overnight. The cells were then washed twice with PBS and stained with 0.005% propidium iodide (PI) at 4 °C in the dark for 30 min. To perform the cell cycle evaluation, the PI-stained cells were analyzed via a BD FACSCalibur cytometer and the data were quantified using the Modfit LT software. Each experiment was repeated three times.

### Inhibitors

Two inhibitors were used in this study: AG1024 (IGF1R inhibitor; Promega, Madison, WI, USA) and LY294002 (PI3K inhibitor; Promega, Madison, WI, USA).

### Migration assay

A Boyden chamber and 8-μm pore size polycarbonate membrane filters (Neuro Probes, Inc., Gaithersburg, MD, USA) were used to assess cell migration. The RSC96 Schwann cells were added to the upper part of chamber, and the bottom chamber was filled with DMEM medium containing 10% FBS. After being incubated overnight, the cells were allowed to migrate through the filters. The cells on the membrane filter were fixed with methanol and Giemsa stain (Sigma, St. Louis, MO, USA). The number of migrated cells was counted with a counting grid in five random fields.

### Western blotting

Protein samples were separated in various percentages of SDS-polyacrylamide gels (8%, 10% or 12% separating gels with 5% stacking gels). After electrophoresis was performed, the proteins were transferred to nitrocellulose membranes. The membranes were blocked with blocking buffer (5% skim milk, 150 mM NaCl, 20 mM Tris-HCl and 0.1% Tween 20) for 1 h at room temperature, washed three times in PBS, and subsequently incubated with the appropriate primary antibodies overnight at 4 °C. Then, the membranes were washed with TBST buffer 3 times and incubated with the secondary antibody for 1 h at room temperature. The membrane data were recorded using a LAS-4000 mini (GE Healthcare Life Sciences).

### siRNA transfection

Double-stranded siRNA sequences targeting PI3K mRNAs were purchased from Dharmacon (Lafayette, CO, USA). The non-specific duplex (scramble) was non-targeting and used as a control. The transfection of siRNA was carried out with a transfection reagent (PureFection™, System Biosciences, Mountain View, CA) according to the manufacturer’s instructions. To assess the gene silencing efficiency, the PI3K protein level was determined using western blot.

### Statistical analysis

Each experiment was performed in triplicate. The results are presented as the mean ± SEM, and the statistical comparisons were performed by Student’s *t* test. Significance was defined at the *p* < 0.05, 0.001 or the 0.0001 level.

## Results

### AOF promotes regenerative signaling of damaged peripheral nerves

**Fig. 1:**
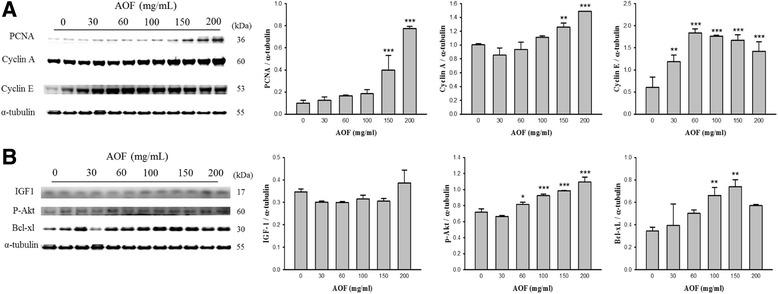
The regeneration of dissected sciatic nerves in the chambers filled with AOF. Sprague-Dawley rats weighing 220 ± 20 g underwent silicone chamber placement. The chambers in the right legs were filled with various concentrations of AOF as indicated. The sciatic nerves from the chamber in rats with surgery were taken. As shown by western blot analyses, AOF treatment upregulated PCNA, cyclin A, cyclin E (**a**) and the IGF-1, p-Akt, Bcl-XL (**b**) in a dose-dependent manner. α-tubulin was used as a loading control

To identify the positive effects of AOF promoting damaged nerve regeneration, the rat sciatic nerves from the chamber treated with AOF were taken by surgery, and the modulation in the expression of proliferation-related proteins was examined. AOF activated the cell cycle as evidenced by increased levels of cyclin A, cyclin E, and proliferating cell nuclear antigen (PCNA) (Fig. 1a). To investigate the role of IGF-1 signaling in AOF-induced nerve proliferation, the activities of IGF-1 signaling in the regenerated nerves were examined, and the observations showed that the protein levels of IGF-1, p-Akt and BCl-xL were increased (Fig. 1b). These findings suggest that AOF promoted nerve cell regeneration.

### Dose-dependent proliferation and survival of RSC96 cells treated with AOF

**Fig. 2:**
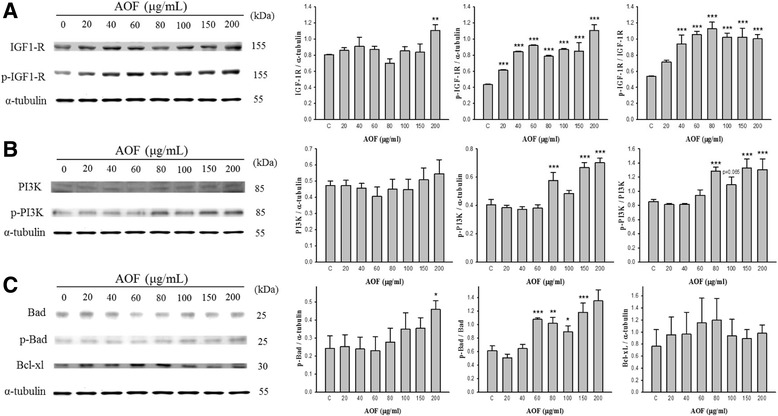
Dose dependence of IGF-1 mediated PI3K signal pathway activation in RSC96 cell treated with AOF extract. Western blot analysis showed that the protein level of IGF-1-related PI3K signal pathway increased in a dose-dependent manner in RSC96 cells treated with AOF (20, 40, 60, 80,100, 150 and 200 μg/mL). α-tubulin was used as a loading control (**a**-**c**)

To evaluate the effect on proliferation of RSC96 Schwann cells, cell proliferation was examined after 24 h of treating with 0, 20, 40, 60, 80, 100, 150 and 200 μg/mL of AOF extract. Western blot analysis revealed that both the survival- and proliferation-related proteins mediated by IGF-1 were markedly increased with AOF treatment (Fig. 2).

Our data demonstrate that IGF-1 expression was markedly increased with AOF treatment. Furthermore, the IGF-1 signaling-related protein expression of the PI3K and Akt were both rapidly induced in a dose-dependent manner.

These findings suggest that AOF potentially promoted Schwann cell proliferation and survival by activating the IGF-1-mediated survival signal mechanism.

### AOF treatment stimulates G_1_ phase cell cycle progression

**Fig. 3:**
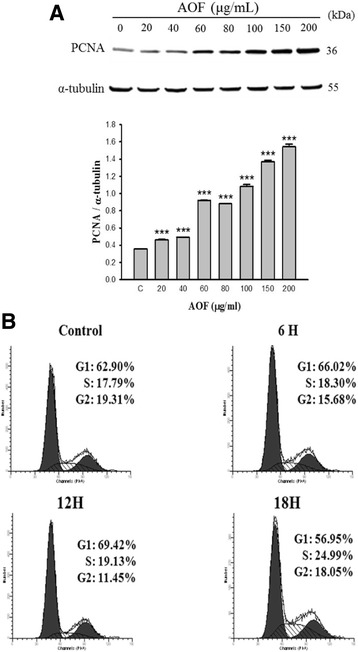
AOF extract induce the expression of PCNA and promote G_1_ progression. RSC96 cells were stimulated for 24 h. **a** As shown by western blot analyses, AOF treatment upregulated PCNA in a dose-dependent manner. *α*-tubulin was used as a loading control. **b** Cell cycle distribution was analyzed using flow cytometry. The proportion of RSC96 Schwann cells were increased significantly in the S phase, but decreased dramatically in the G1 phase at 12 h–18 h

We further investigated whether AOF stimulated the cell proliferation and related mechanisms. The levels of PCNA expression and the cell cycle distribution were determined using western blot and flow cytometry, respectively. The results showed that PCNA levels increased in a dose-dependent manner (Fig. 3a).

Representative examples of the flow cytometry histograms showed that the cell cycle distribution of RSC96 Schwann cells were altered apparently in the time-course AOF treatment experiments. Furthermore, the proportion of RSC96 Schwann cells in the S phase increased significantly from 17.79% to 24.99%, and the proportion of cells in the G1 phase decreased dramatically from 69.42% to 56.95% at 12 h–18 h (Fig. 3b). These results suggest that AOF likely facilitated cell proliferation by accelerating the cell cycle progression.

### AOF increases the level of cell cycle proliferative proteins

**Fig. 4:**
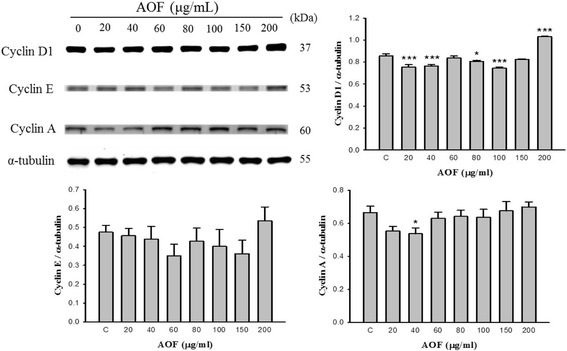
AOF extract induces the expression of proteins involved in the cell cycle in a dose-dependent manner. RSC96 cells were stimulated with AOF for 24 h. The protein expressions of cell cycle regulatory proteins were determined by western blot analysis. Treatment with AOF upregulated cyclinD1, cyclinE and cyclinA in a dose-dependent manner. α-tubulin was used as a loading control

It is known that cell cycle progression is controlled by a regulatory network; cyclins are one of the important regulatory molecules involved in cell cycle control. To evaluate how AOF regulates the cell cycle to facilitate RSC96 Schwann cell proliferation, a western blot was performed to characterize the key proteins of cell cycle regulation (Fig. 4). In addition, the AOF treatment induced the up-regulation of cyclin A expression in a dose-dependent manner. These results implied that AOF increases the level of cyclins, further stimulating the proliferation of RSC96 Schwann cells.

### RSC96 Schwann Cell proliferation enhanced by AOF was associated with IGF-1 and PI3K/Akt signaling

**Fig. 5:**
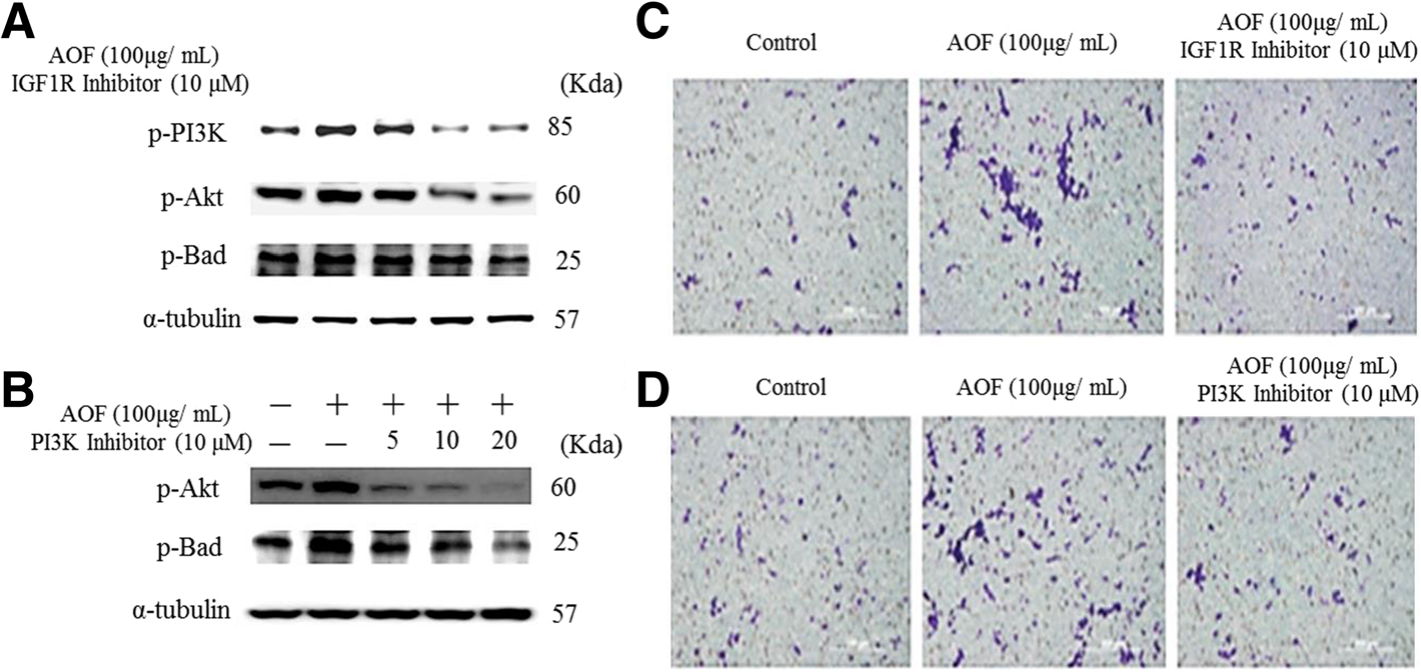
AOF extract effects on Schwann cell proliferation was IGF-1signaling dependent. RSC96 cells were pretreated with IGF1R inhibitor (**a**) or PI3K inhibitor (**b**) for 1 h, and then treated with 100 μg/ml AOF extract for 24 h. Western blot analysis showed that AOF-induced the up-regulation of p-PI3K, p-Akt and p-Bad which was markedly suppressed when treated with either AG1024 (IGF1R inhibitor; Promega, Madison, WI, USA) or LY294002 (PI3K inhibitor; Promega, Madison, WI, USA) inhibitor.α-tubulin was used as a load control. After incubation with AOF extract, migration assay was performed using Boyden chambers treated with IGF1R inhibitor and PI3K inhibitor, respectively (**c** and **d**). The number of migrated cells was much more in the cells treated with AOF extract, but the migration effect was blocked when treated with IGF1R inhibitor and PI3K inhibitor

**Fig. 6:**
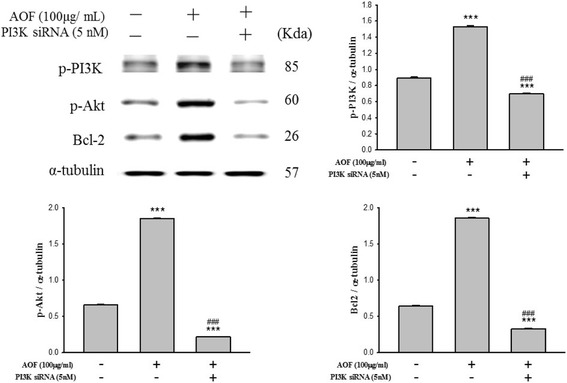
PI3K knockdown inhibited AOF extract-induced survival and proliferation. Schwann cell was transiently transfected with 5 nM PI3K siRNA for 1 h prior to treatment. After incubation with 100 μg/mL AOF extract for 24 h, cells were harvested and analyzed by immunoblotting. *α*-tubulin was used as a loading control

Because a relationship between AOF-triggered IGF-1 signaling with cell proliferation was found in RSC96 Schwann cells, we explored whether the activation of the IGF-1R and/or the PI3K signaling pathway is necessary for the AOF-induced cell proliferation. RSC96 Schwann cells were pretreated with AG1024 and LY294002 as specific inhibitors of the IGF-1R and PI3K/Akt, respectively, followed by incubation with 100 μg/mL AOF extract for 24 h. This study demonstrated that the AOF-induced up-regulation of p-PI3K, p-Akt and p-Bad was markedly suppressed by the inhibitor treatment (Fig. 5a and b). To further confirm these results, we used the siRNA-induced knockdown of p-IGF-1R and p-PI3K proteins (Fig. 6). The results of western blot analyses demonstrated decreased expression of p-PI3K, p-Akt and p-Bad.

These results implied that AOF extract-induced Schwann cell proliferation occurs by the activation of the IGF-1R-mediated survival pathways.

## Discussion

**Fig. 7:**
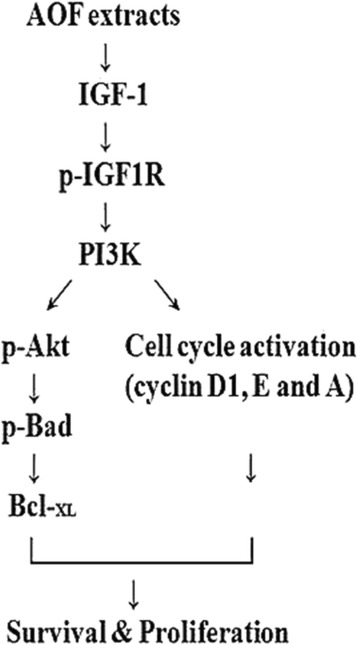
Schematic model of the survival and proliferative effects of AOF extract on RSC96 Schwann cell. Stimulation of Schwann cell with AOF extract activate IGF-I signaling, leading to upregulation of the PI3K/Akt pathway and activation of the cell cycle regulatory proteins cyclin D1, E and A, resulting in the survival and proliferation of RSC96 Schwann cell

Previous reports have proposed the well model for assessing the capacity of regenerating neurons that are filled with a mixture of AOF extract and Schwann cells in a silicone rubber chamber for bridging a 15-mm rat sciatic nerve gap [14–18]. It has also been reported that Schwann cells are vital requirements in the injured nerve area, which could trigger nerve regeneration by trophic factors and the generation and release of adhesion molecules [19, 20]. AOF is a widely used traditional Chinese medicine, which has been reported to have a potential neuroprotective effect [8–12]. However, for considering the clinical application of AOF, it is necessary to determine the mechanism by which AOF-extract promotes the survival and proliferation of Schwann cells. The mechanism involved in the coordinated events is presented in Fig. 7.

Neurotrophic factors, including IGFs, are a set of growth factors that influence the growth and regeneration of neurons [21–25]. According to recent reports, IGF-1 plays an important role in cell survival and Schwann cell proliferation [4, 26]. Regenerating peripheral nerves show high IGF-1 expression in their advancing growth cone. The IGF-1 expression activates the PI3K/Akt signaling pathway and is associated with the proliferation of Schwann cells and regeneration of peripheral nerves [27–32].

AOF extract also induced cell proliferation via activating the PI3K/Akt signaling pathway in RSC96 cells. The PI3K signaling induced by IGF-1 attenuates Schwann cell apoptosis by caspase activation and is also known to facilitate the transition of cell cycle from the G_1_ to S phase [31, 33]. Inhibition of PI3K effectively inhibits the IGF-1-induced anti-apoptotic and neuro-protective effects, and exhibits the significant role of PI3K in the induced survival of Schwann cells [29, 30]. Bioactive compounds that induce the up-regulation of IGF-1 can potentially activate the PI3K/Akt signaling and lead to modulating the expression of regulatory proteins (Bcl-2, p-Bad, PCNA and cyclins) to favor Schwann cell survival and proliferation during nerve regeneration. We show a beneficial effect in the up-regulation of the IGF-1-related survival pathway and the stimulation of cell proliferation in cultured RSC96 cells following AOF treatment.

Cell proliferation is regulated at various levels, and the G1/S cell cycle transition is one of the most important requirements for DNA replication and mitosis.

Progression through the G_1_ phase into the S phase requires cyclin D and cyclin E activity; whereas, Cyclin A activity is required for the DNA replication of S phase and mitosis initiation [34–36]. Our current results showed that the G1/S transition of cell cycle progression was induced by AOF extract treatment (Fig. 3b). The time course study revealed that the AOF extract treatment is effective in cyclin A up-regulation, subsequent promotion of DNA replication and the increasing cell number in the S phase, which eventually results in cell proliferation. However, the expressions of cyclin D1 and cyclin E did not show any significant change. In certain cells, such as hematopoietic cells, the IGF expression acts as an inhibitor of cell death [37, 38]. However, IGF-1 has also been shown to stimulate cell cycle progression via G_1_ or the G_0_/G_1_ transition in cultured fibroblasts and mammary epithelial cells [39–41]. We also provide data demonstrating that the cell cycle is driven by cyclins with corresponding changes in IGF-1 levels. AOF-induced alterations of the cell cycle in RSC96 cells may serve as critical determinants for nerve cell proliferation and IGF-1-related cell survival. Despite the fact that we cannot exclude other minor candidates involved in the related pathways, we clearly showed that certain proteins of the IGF-1-related signal pathway were up-regulated following AOF extract treatment.

Systematic survey on literatures shows that over 80 chemical constituents which were identified from *A. oxyphylla* including diarylheptanoids, flavonoids, polyphenols, sesquiterpenes, steroids, volatile oil and their glycosides, etc. [42–48]. Emerging studies evaluated that the abundance of nine secondary metabolites differentially concentrated in seeds and fruit capsules of *A. oxyphylla*, including sesquiterpenes (e.g., nootkatone), diarylheptanoids (e.g., oxyphyllacinol and yakuchinone A and B) and flavonoids (e.g., chrysin, izalpinin, tectochrysin, kaempferide and apigenin-4′,7-dimethylether) [49, 50]. Protocatechuic acid (PCA), one of the major metabolite of complex polyphenols, which has been identified in the kernels of AOF [9, 51, 52], it also has been reported that PCA process positive effect on antioxidant, anti-inflammatory, and antiapoptosis [8–11, 53]. Our recent studies revealed that PCA promoted RSC96 Schwann cells regeneration and migration by activating ERK1/2, JNK1/2 and p38 MAPK pathways [54]. It also been reported that PCA effectively promoted RSC96 cell survival and proliferation by activating IGF-IR-PI3K-Akt signaling [55]. The protective effects associated with the chemical constituents of PCA correlates with the pharmacological activities of AOF as defined in the present study.

## Conclusion

In summary, it was found that AOF extract significantly increases the IGF-1-related PI3K/Akt pathway in RSC96 Schwann cells, and upregulates the cell cycle regulatory proteins cyclin A, cyclin D1 and cyclin E. Therefore, AOF extract might be effective for RSC96 Schwann cell proliferation and survival.

## Abbreviations

AKT: Protein kinase B
AOF: Alpinia oxyphylla Miq
Bad: Bcl-2-associated death promoter
Bcl-2: B-cell lymphoma-2
Bcl-xL: B-cell lymphoma-extra large
DMEM: Dulbecco’s modified eagle’s medium
FBS: Fetal bovine serum
IGF-1: Insulin-like growth factor-1
IGF-1R: Insulin-like growth factor-1 receptor
PBS: Phosphate buffered saline
PCNA: Proliferating cell nuclear antigen
PI: Propidium iodide
PI3K: Phosphoinositide 3 kinase
SDS-PAGE: Sodium dodecyl sulfate polyacrylamide gel electrophoresis
